# Bayesian optimization of distributed neurodynamical controller models for spatial navigation

**DOI:** 10.1016/j.array.2022.100218

**Published:** 2022-07-15

**Authors:** Armin Hadzic, Grace M. Hwang, Kechen Zhang, Kevin M. Schultz, Joseph D. Monaco

**Affiliations:** a The Johns Hopkins University Applied Physics Laboratory, Laurel, 20723, MD, USA; b Kavli Neuroscience Discovery Institute, Johns Hopkins University, Baltimore, 21218, VA, USA; c Department of Biomedical Engineering, Johns Hopkins University School of Medicine, Baltimore, 21205, MD, USA

**Keywords:** Bayesian optimization, Multi-agent control, Swarming, Dynamical systems models, Spatial navigation, UMAP

## Abstract

Dynamical systems models for controlling multi-agent swarms have demonstrated advances toward resilient, decentralized navigation algorithms. We previously introduced the NeuroSwarms controller, in which agent-based interactions were modeled by analogy to neuronal network interactions, including attractor dynamics and phase synchrony, that have been theorized to operate within hippocampal place-cell circuits in navigating rodents. This complexity precludes linear analyses of stability, controllability, and performance typically used to study conventional swarm models. Further, tuning dynamical controllers by manual or grid-based search is often inadequate due to the complexity of objectives, dimensionality of model parameters, and computational costs of simulation-based sampling. Here, we present a framework for tuning dynamical controller models of autonomous multi-agent systems with Bayesian optimization. Our approach utilizes a task-dependent objective function to train Gaussian process surrogate models to achieve adaptive and efficient exploration of a dynamical controller model’s parameter space. We demonstrate this approach by studying an objective function selecting for NeuroSwarms behaviors that cooperatively localize and capture spatially distributed rewards under time pressure. We generalized task performance across environments by combining scores for simulations in multiple mazes with distinct geometries. To validate search performance, we compared high-dimensional clustering for high- vs. low-likelihood parameter points by visualizing sample trajectories in 2-dimensional embeddings. Our findings show that adaptive, sample-efficient evaluation of the self-organizing behavioral capacities of complex systems, including dynamical swarm controllers, can accelerate the translation of neuroscientific theory to applied domains.

## 1. Introduction

Collective biological behaviors of animal groups, including swarming, flocking, and schooling behaviors [1–6] have long inspired robotics and computer science research into problems of decentralized control and coordination for autonomous groups of artificial agents [7–12]. In particular, advancing the autonomous spatial capabilities of multi-agent swarm control has been a key objective of simulation studies and analyses of artificial swarms based on dynamical systems models [13]. Complementarily, the impressive recent progress of artificial intelligence based on deep learning [14] has demonstrated the importance of adopting key biological inspirations from neuroscience and the brain. However, it has been unclear how to integrate complex temporal features of brain dynamics thought to support crucial mechanisms of neural computation [15]. Thus, addressing critical questions in autonomous robotics and artificial intelligence may depend on efficient exploration and optimization of dynamical systems models with complex interactions among many units. In both domains, major gaps in state-of-the-art capabilities are highlighted by tasks involving autonomous spatial navigation and foraging [16–19] in complex, novel, or changing environments.

Bayesian optimization provides a probabilistic framework for adaptive, sample-efficient optimization of ‘black box’ models with moderate dimensionality (up to ~20 parameters) and expensive sample evaluations. In this framework, a task-dependent objective function signifies the output performance of the complex underlying model, and the optimizer traces parameter-space trajectories of candidate points from acquisition functions operating on a simpler surrogate model. The typical surrogate model is a Gaussian process that populates the parameter space of interest with multivariate normal distributions and which serves as a prior distribution for candidate-point updates [20, 21]. Bayesian optimization with Gaussian process surrogate models has enabled applications including the hyperparameter tuning and optimization of evolutionary algorithms, multi-modal functions, robotic controllers, and other complex systems [22–27].

The collective behavioral states of some swarming models are tractable to linear analysis of stability, density, and clustering properties [28–32]. However, for dynamical systems that preclude such analysis due to nonlinearity, nonstationarity, stochasticity, or other complications, the computational budget for parameter exploration or optimization with simulation-based samples is a limiting factor for translation to engineered designs. Indeed, standard methods based on gradient descent have two main drawbacks in this context: they can discover local optima, but resist exploration of system behaviors for other purposes; and their basic operation is massively sample-inefficient, which can be prohibitive for expensive simulation-based sample evaluations. Moreover, emergent collective behaviors like swarming outstrip conventional agent-based learning methods based on the restrictive action and policy spaces of reinforcement learning, particularly for uncertain, changing, or open-ended tasks.

We previously introduced the *NeuroSwarms* framework for modeling emergent high-level navigation and foraging in a brain-inspired multi-agent metacontroller [33–35]. NeuroSwarms addressed decentralized, distributed control by analogy to neural circuit dynamics, including oscillations [36–39] and attractors [40–42], and associative synaptic plasticity [43] related to rodent spatial cognition; the resulting collective behaviors of NeuroSwarms models included swarming, patroling, and goal-finding in simulated maze environments with complex, irregular, or fragmented geometry [34]. These behaviors enabled NeuroSwarms to complete cooperative multiple reward-capture tasks without pretraining across distinct environments [34]. However, the nonlinearities inherent in NeuroSwarms’ oscillatory phase-coupled self-organization precluded analytic approaches to global identification, exploration, or optimization of system behaviors. Thus, this class of dynamical systems model can provide insights into key aspects of brain structure and function that may inspire theoretical advances as well as new directions for systems engineering designs. This insight depends crucially on devising a task-dependent objective function that can guide the efficient discovery of system behaviors and optimal performance. In this paper, we demonstrate that Bayesian optimization can utilize such an objective function to efficiently and usefully find paths through otherwise prohibitive model spaces. In particular, we show that a neurodynamical controller model with emergent properties can be characterized and tuned using Bayesian optimization with Gaussian process surrogate models.

## 2. Models and methods

### 2.1. NeuroSwarms model

Monaco et al. (2020) [34] introduced the NeuroSwarms framework and described a model implementation with 300 agents; baseline wall-avoiding, momentum-carrying motion-vector updates; maze environments whose geometry occluded agents’ line-of-sight; interagent communication between mutually visible agents; cosine-coupling of internal phase variables driving interagent attraction and repulsion; and 9 key dynamical parameters (Table 1) that had required intensive manual fine-tuning to balance swarming and reward capture.

### 2.2. Bayesian optimization

**Fig. 1.**
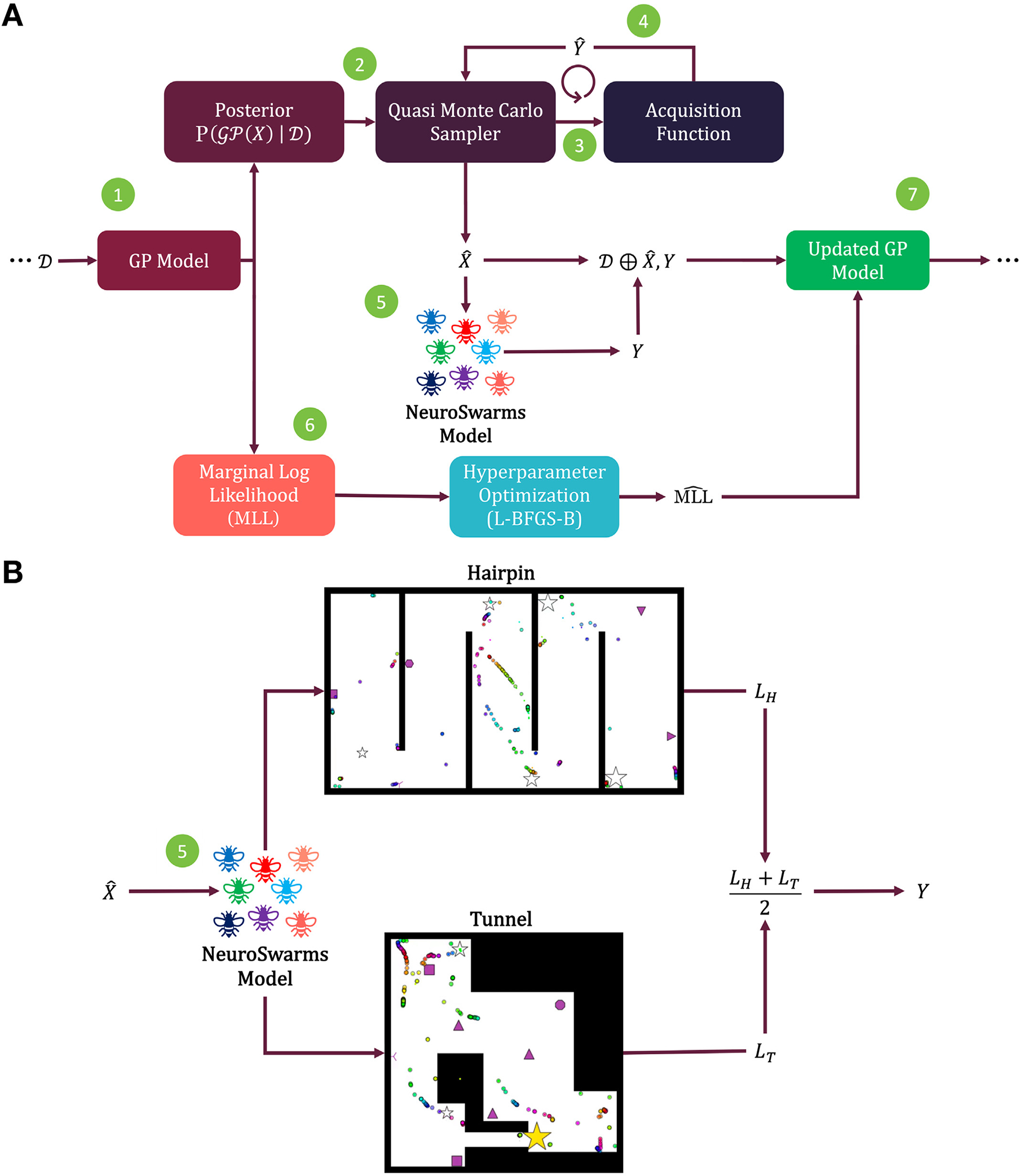
Computation flow for optimization and simulation-based sampling. *A*, Step 1: The posterior distribution is computed from the Gaussian process surrogate model (GP Model) based on the training data 𝒟. Step 2: The acquisition function’s Quasi Monte Carlo sampling process uses the posterior distribution to select new candidate parameters $\hat{X}$ (Step 3) based on the acquisition function’s estimated objective function value $\hat{Y}$ (Step 4). Step 5: The NeuroSwarms model [33,34] is simulated with candidate parameter points $\hat{X}$ to generate the observed objective value *Y* (see *B*). Step 6: The initial Gaussian process model’s marginal log-likelihood (MLL) is then calculated and used to optimize the Gaussian process using the L-BFGS-B algorithm [47]. Step 7: The resulting 𝒟 (from Step 5) and MLL (from Step 6) update the Gaussian process model for the next iteration of the outer loop. *B*, Flow diagram of simulation-based candidate-point evaluation. For each sample (see Step 5 in *A*), the optimizer executes play-throughs in both the Hairpin (top) and Tunnel (bottom) maze environments. The sample’s objective value *Y* is computed as the average of the respective loss values *L*_*H*_ and *L*_*T*_ (Eq. (3)).

Bayesian optimization constructs and performs sequential optimization on a surrogate model that represents the objective performance of a more complex model [44–46]. Learning surrogate models can be beneficial if directly optimizing a complex model is not computationally tractable given resource constraints. These surrogate models can then be deployed to predict the performance of the underlying model at untested parameter points without requiring a full model simulation of those parameter values (Fig. 1).

We implemented Bayesian optimization with surrogate models defined as Gaussian processes [20,48,49]. Gaussian processes are parametric models that iteratively learn a probabilistic mapping f:X↦ℝ such that the density estimate p(*y*_*i*_|**x**_*i*_) = *f*(**x**_*i*_*, y*_*i*_), where $X\subseteq {\mathbb{R}}^{p}$ is the bounded parameter subspace being optimized, $x_{i}\in X$ is a parameter point, and $y_{i}\in \mathbb{R}$ is an objective function output value [21,50,51]; e.g., *p* = 9 NeuroSwarms parameters in this paper. Thus, the underlying ‘black box’ objective function *f*_true_ is assumed to be distributed according to a Gaussian process,

$$f_{\text{true}}\sim 𝒢{𝒫}_{\mu ,k}(X),$$

where *μ*(·) and *κ*(·) are mean and covariance kernels applied to an input parameter set, X⊂X. The posterior distribution of a *q*-sized batch of candidate points $\hat{X}=\left\{{\hat{x}}_{1},\ldots ,{\hat{x}}_{q}\right\}$ conditioned on the observed training data $𝒟={\left\{\left(x_{i},y_{i}\right)\right\}}_{i=1}^{n}$ takes the form of a *p*-dimensional multivariate normal distribution, i.e., $\text{P}(𝒢𝒫(X)|𝒟)\sim {𝒩}^{p}(\mu (X),k(X))$.

### 2.3. Acquisition functions

Bayesian optimization relies on acquisition functions to provide the candidate parameter points that navigate the underlying model space. Acquisition functions define a strategy to manage the trade-off between exploring the parameter space and exploiting regions that yielded improvement for previous samples [52]. An acquisition function can be evaluated on the Gaussian process posterior P(𝒢𝒫(X)∣𝒟) by averaging a set of Monte Carlo (MC) samples, e.g.,

(1) $${\hat{\alpha }}_{n}(X;𝒟)=\frac{1}{n}\sum_{i=1}^{n}a\left(\varepsilon_{𝒟}^{i}(X)\right),$$

where *n* is the sample count and *a*(·) is the net utility function providing objective function output. Thus, ${\hat{\alpha }}_{n}$ is an expectation of posterior samples $\varepsilon_{𝒟}\sim \text{P}(𝒢𝒫(X)|𝒟)$. We study a pair of MC-based acquisition functions: 𝑞-Expected Improvement (qEI) [53] and Noisy 𝑞-Expected Improvement (qNoisyEI) [54]. We compare qEI and qNoisyEI to random sampling of candidate parameters. First, similar to ${\hat{\alpha }}_{n}$ (Eq. (1)), qEI calculates an expectation over posterior samples,

$$\text{qEI}(X)\approx \frac{1}{n}\sum_{i=1}^{n}\overset{q}{\underset{j=1}{\max}}{\left[\varepsilon_{j}^{i}-Y^{*}\right]}_{+},$$

where [·]_+_ indicates linear rectification and 𝑌* is the best observed objective function value. Thus, qEI estimates a noise-free expected improvement of the posterior with respect to the best value. Second, qNoisyEI approximates improvement relative to the expected best objective value conditioned on the observed MC sampling history *ε*_obs_ within each batch [55]; simplistically, the constrained batch-sampling performed by qNoisyEI [54,56] approximates

$$\text{qNoisyEI}(X;𝒟)\approx \frac{1}{n}\sum_{i=1}^{n}\overset{q}{\underset{j=1}{\max}}{\left[\varepsilon_{j}^{i}-\max\varepsilon_{\text{obs}}\right]}_{+},$$

but more detailed treatments of this complex optimization problem provide critical analyses and caveats (cf. [54–56]).

Throughout our study, Bayesian optimization with any of the three acquisition functions employed 512 MC samples, 30 training epochs (with a batch size of 3), and 8 random training samples to initialize the Gaussian process surrogate model.

### 2.4. Objective function

We constructed an objective function to evaluate the performance of the example NeuroSwarms model [34] in a time-pressured cooperative foraging task. The objective function quantifies how quickly the swarm of agents collectively capture several spatially distributed rewards in a given maze. Let *n*_cap_(*t*) be the cumulative number of cooperatively captured rewards by time *t*. A reward is captured if, at any timestep, at least *n*_*s*_∕*n*_*r*_ agents were simultaneously colocated within a defined radius from the reward, where *n*_*s*_ = 300 agents and *n*_*r*_ = 3 and 5 rewards in the Tunnel and Hairpin mazes, respectively. For a given simulated play-through, this objective function can be expressed as a loss which is updated at every timestep until all rewards are captured,

(2) $$L=-t\;/\left(n_{t}n_{\text{cap}\;}(t)+1\right),$$

where *n*_*t*_ is the total number of time steps. The agent group’s behavior is time-pressured by *t* growing continuously until all rewards are captured. If the swarm is not able to capture all the rewards in the environment, *t* will be set to the maximum number of timesteps allowed for the simulation *n*_*t*_ and the loss will reflect the number of missed rewards. Loss values range from [−1, 0], with better task performance closer to zero.

To account for the generalizability of spatial task performance across distinct environmental geometries, each simulation-based sample constitutes play-throughs of both the Hairpin and Tunnel mazes, respectively providing loss values *L*_*H*_ and *L*_*T*_ as calculated in Eq. (2) (see Fig. 1B). Thus, the generalized performance at a given parameter point **x**_*i*_ is indicated by the objective value *Y*, computed as the average

(3) $$y_{i}\left(x_{i}\right)≐Y=\frac{L_{H}\;+\;L_{T}}{2}.$$

### 2.5. Gaussian process training

The means and variances of the Gaussian process surrogate model are updated with each sample evaluation to reflect the expected values and uncertainty, respectively, of the underlying model’s performance. We use the Bayesian optimization library BoTorch [51] to implement the outer loop of surrogate model training based on iteratively updating a updating a Gaussian process following initialization with sample data 𝒟. The posterior distribution P(𝒢𝒫(X)∣𝒟 is then sampled from a batched MC sampling process using an acquisition function to determine the candidate parameter points $\hat{X}$ from the subspace bounded by the ranges listed in Table 1. The candidate points are selected based on predictive estimates of utility value $\hat{Y}$ (Fig. 1A) and evaluated by simulating the NeuroSwarms model to generate loss values (Eq. (2)) and objective function output *Y* (Eq. (3)) (Fig. 1B). Lastly, the resulting $\left(\hat{X},Y\right)$ tuple is appended to training data 𝒟 to update the Gaussian process for the next iteration.

The surrogate model hyperparameters were tuned by first computing the marginal log-likelihood (MLL) of the Gaussian process applied to observed parameters *X* and fitting hyperparameters with the limited-memory Broyden–Fletcher–Goldfarb–Shanno algorithm with simple bounds (L-BFGS-B) [47]. The fitting process provides an updated MLL for the next optimization step.

#### 2.5.1. Convergence metrics

This hyperparameter tuning process described above was repeated until convergence according to two metrics: maximum posterior variance and minimum candidate dissimilarity. First, maximum posterior variance for training epoch *M* was computed following

$$\max\mathrm{Var}\left(\text{P}\left(𝒢𝒫\left(x_{M}\right)|{𝒟}_{M}\right)\right)$$

to indicate whether the Gaussian process’ posterior variance was no longer increasing and that training should cease. Second, minimum candidate dissimilarity measures the stabilization of candidate selection as an inverse cosine similarity; i.e., we calculated the metric following

$$\overset{M-1}{\underset{i=1}{\min}}\left[1-\frac{x_{i}\;\cdot \;x_{M}}{\left\|x_{i}\right\|\;\cdot \;\left\|x_{M}\right\|}\right]$$

to confirm whether epoch *M* selected for similar neighborhoods of parameter points as in previous training epochs. These convergence metrics determined hyperparameter convergence and enabled the resulting Gaussian process surrogate model to efficiently adapt to the NeuroSwarms parameter space.

### 2.6. Parameter visualization

The low-dimensional representations produced by the uniform manifold approximation and projection (UMAP) [57] result from a locality-preserving embedding that serves to spatially cluster higher-dimensional vectors such as *p*-dimensional parameter points. A 2D UMAP projection allows these point clusters to be simply visualized as images or scatter plots, for which the *x*-axis and *y*-axis constitute an arbitrary coordinate frame. For UMAP scatter plots, as in Figs. 3 and 6, the marker for each point can be colored for convenient visual inspection of associated values, including vector elements or computed output. We use this visual clustering to qualitatively inspect the parameter-dependence and structure of the Gaussian process surrogate model by selecting a UMAP data point with, e.g., high performance indicated by its loss value *y*_*i*_ (Eq. (3)), and assessing that point’s other values in the context of its location and neighborhood relative to UMAP-based clusters.

## 3. Results and discussion

### 3.1. Overview

We demonstrate Bayesian optimization methods (see Section 2.2) for tuning the parameters of a neuroscience-inspired swarming model, NeuroSwarms [33,34,39] (see Section 2.1), to find cooperative foraging behaviors for capturing multiple rewards in distinct maze environments under time pressure (see Section 2.4). We train Gaussian process surrogate models (see Section 2.5) to characterize the NeuroSwarms parameter space using noise-free (i.e., qEI) and observed sampling history-dependent (i.e., qNoisyEI) acquisition functions (see Section 2.3). Then we show how the locality-preserving dimensionality reduction provided by UMAP embeddings (see Section 2.6) can be used to evaluate the surrogate model and identify system behaviors.

### 3.2. Training the surrogate model for swarming performance

Small variations in the *p* = 9 dynamical NeuroSwarms parameters (Table 1) can substantially impact collective behaviors. Optimal parameters that allow NeuroSwarms models to accomplish generalized cooperative foraging may not be limited to a single set of parameters due to the complexity and potential degeneracy of emergent collective behaviors in a distributed multi-agent system. Thus, we constructed a simple time-pressured objective function to measure the progress of reward-capture (Section 2.4) and guide Bayesian optimization using Gaussian process surrogate models (Fig. 1A). We utilized acquisition functions to sample candidate parameter points and optimize the Gaussian process’ predictive performance compared to observed NeuroSwarms simulations (Section 2.5). We evaluated the surrogate models in two environments for each sample: a Hairpin maze and a Tunnel maze (Fig. 1B). By simultaneously assessing mazes with distinct geometries, the surrogate model optimization was allowed to find swarming and navigational dynamics resulting in time-efficient cooperative foraging that may generalize across environments.

**Fig. 2.**
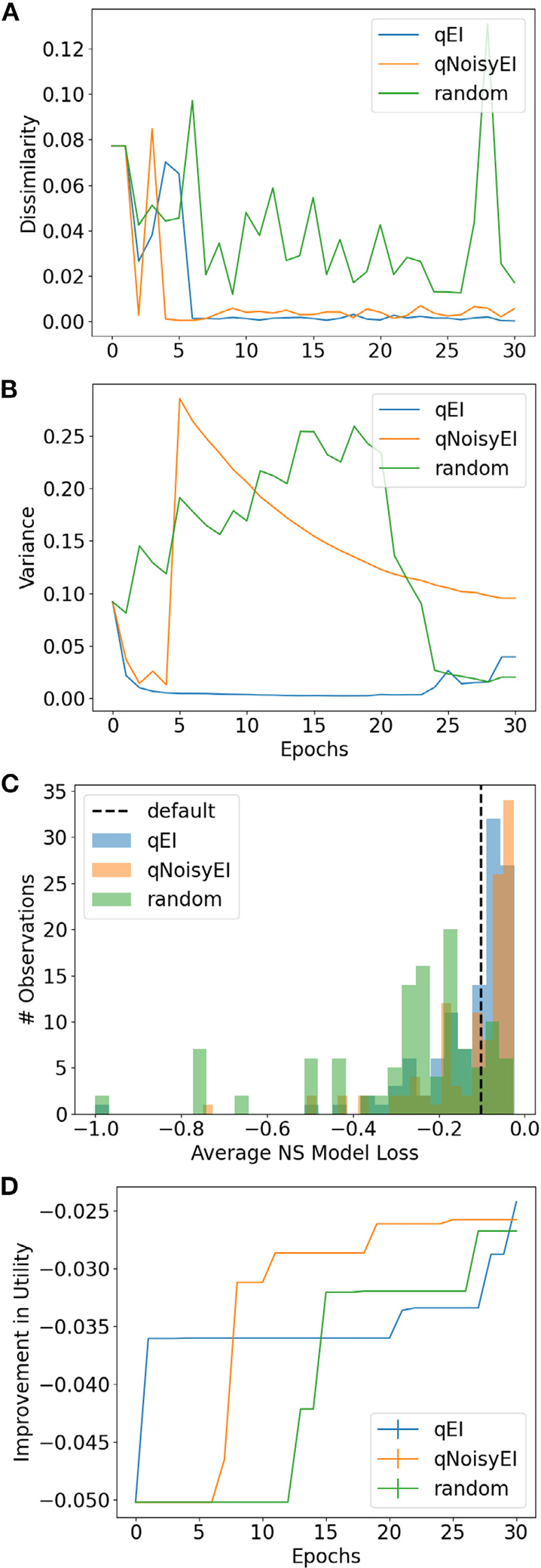
Convergence metrics and objective function values for acquisition functions across training. *A*+*B*, Training convergence metrics: minimum candidate dissimilarity (*A*) and maximum posterior variance (*B*). *C*+*D*, The training performance of Gaussian process models based on the qEI and qNoisyEI acquisition functions, compared to a baseline of random sampling, was quantified by objective function values shown as histograms of losses for the sampled parameter trajectories (*C*) and as the improvement in best observed values (*D*), where values closer to 0 indicate better performance (Eq. (2)) in the time-pressured cooperative foraging task.

We started training with an initial set of 24 randomly selected parameter points with corresponding simulation results. Each Gaussian process was trained by an acquisition function for selecting candidate points: *q*-batched Expected Improvement (qEI), *q*-batched Noisy Expected Improvement (qNoisyEI), or random parameter sampling (Section 2.3). Gaussian process modeling and training was implemented using BoTorch [51] and optimized with 512 MC samples over 30 training epochs (Section 2.5). We verified that the EI-based acquisition functions converged based on metrics of minimum candidate dissimilarity and maximum posterior variance (Section 2.5.1). The EI-based acquisition functions approached zero dissimilarity during training (Fig. 2A). Similarly, the maximum posterior variance for each surrogate model had converged by the end of training (Fig. 2B).

We evaluated how effective each acquisition function was at finding regions of the parameter space that optimize the NeuroSwarms objective function (Eqs. (2) and (3)). Both qEI and qNoisyEI discovered more parameter points with high-performance values than random sampling (Fig. 2C). Both random sampling and the default parameters from Monaco et al. (2020) [34] were outperformed by the EI-based acquisition functions. Thus, qEI and qNoisyEI demonstrated the strongest utility improvement of best observed values during training as the NeuroSwarms parameter space was learned by the corresponding surrogate models (Fig. 2D).

### 3.3. Evaluating UMAP-clustering of selected parameters

**Fig. 3.**
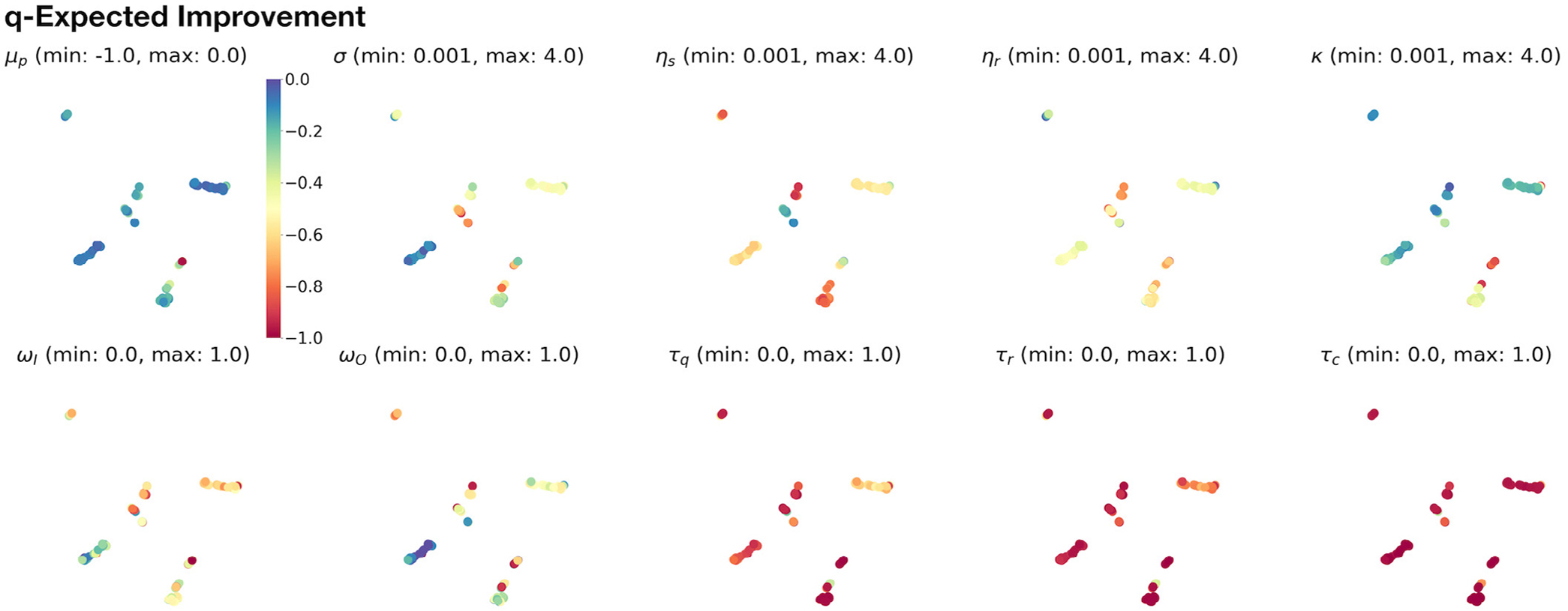
UMAP-clustered parameter points selected by the noise-free qEI acquisition function. The dimensional reduction computed by the UMAP transformation (Section 2.6) preserves locality of neighboring parameter points. As a result, high-dimensional clusters can be revealed by scatter plots of 2D UMAP data. Each of the 10 scatter plots shows the same UMAP projection of qEI-sampled parameter points, using the same (arbitrary) 2D coordinate frame. In the first plot (top, left), the color of each point indicates the expected posterior mean of the trained Gaussian process surrogate model according to the colorbar legend to the right of the plot; e.g., a group of adjacent blue points reflects a high-performing cluster of NeuroSwarms parameters. The top-left colorbar additionally serves to provide a reference for how colors are mapped to the respective value ranges (i.e., [min, max]) specified in the label above the remaining *p* = 9 plots. These 9 plots show the individually sampled parameter values (cf. Table 1) associated with each UMAP point.

Understanding the results of the above Bayesian optimization process requires a visual representation of the parameter space, yet it can be challenging to represent data with >3 dimensions. We considered that visualizing parameter points in lower dimensions could facilitate the discovery of critical surrogate model structures, including clusters of high-performing parameters that potentially yield distinct behavioral solutions to the cooperative foraging task. Thus, we used UMAP (Section 2.6) to reduce sets of 9-dimensional NeuroSwarms parameters (Table 1) into locality-preserving 2D representations. For qEI-selected parameters, we assigned colors to the resulting 2D UMAP-clustered data points according to posterior mean estimates of objective values (top, left plot) or individual parameter values (Fig. 3). The resulting visual representation in Fig. 3 shows where the highest utility (i.e., best posterior mean estimate of objective value) data points cluster into groups based on the values of NeuroSwarms parameters.

Given that qEI demonstrated the largest utility improvement (Fig. 2D) and consistently identified high-performing parameters (Fig. 2C), we consider its UMAP representation for further analysis. The qEI-based parameter samples formed two clusters of data points with the highest utility (Fig. 3). In the (top, left) posterior mean plot, we selected one of these points from the lower, left cluster and matched it with the numerical values of its associated parameters, which we subsequently evaluated in NeuroSwarms simulations.

**Fig. 4.**
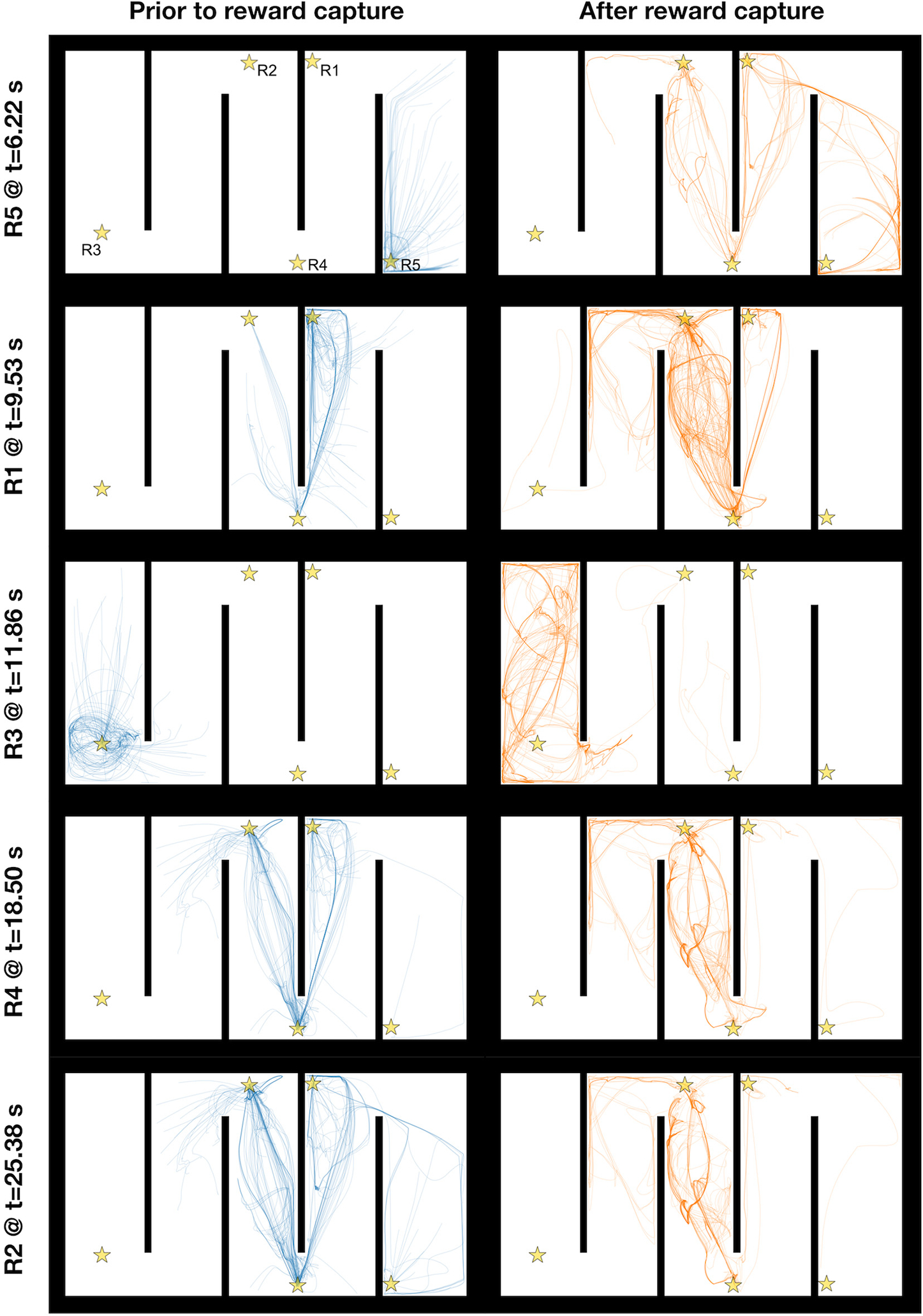
NeuroSwarms trajectories depicting reward capture in the Hairpin maze. The Hairpin maze presents a large, fragmented arena to assess the swarm’s foraging performance given the uncertain localization inherent in environments with symmetrically repeating geometric patterns. Five reward goals are spatially distributed at maze locations indicated by gold stars (R1–R5, top-left maze plot). The 10 maze plots show segments of spatial trajectories traced out by NeuroSwarms agents during a sample simulation. Maze plots on the left show agent paths (blue traces) from either the beginning of the simulation or the most recent previous reward capture to the time of the reward capture indicated by the text label to the left of the plot. Traces are shown for only those agents that contributed to cooperative capture of the given reward (see Section 2.4). Conversely, maze plots on the right show agent paths (orange traces) from the time of reward capture until the end of the simulation. From top to bottom, each row presents a pre-capture and post-capture pair of swarm trace plots in the order in which rewards were captured in the simulation. Individual traces are translucent; thus, the degree to which the trajectories of multiple agents superposed upon the same observed paths is indicated by the relative saturation of the trace color. As a result, visual inspection yields information about the swarming and reward-approach dynamics with respect to the spatial convergence and divergence of agents over time.

**Fig. 5.**
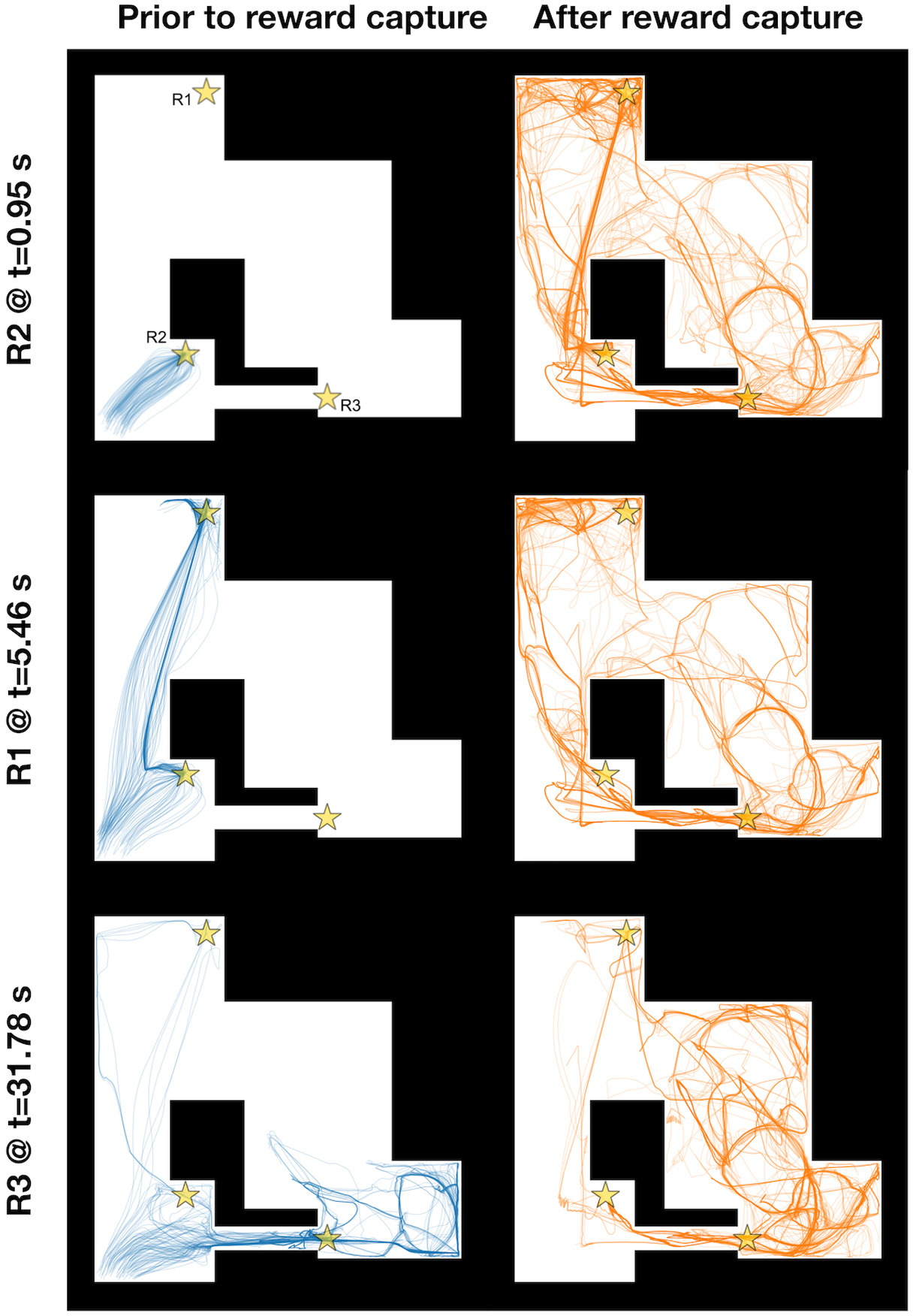
NeuroSwarms trajectories depicting reward capture in the Tunnel maze. The Tunnel maze presents an irregular arena to assess the swarm’s foraging performance given a loop-like environment with substantial geometric occlusion of visibility and passageways with large vs. constrictive (e.g., the eponymous ‘tunnel’ connecting the Southwest to the Southeast quadrants) apertures. Three reward goals are spatially distributed at maze locations indicated by gold stars (R1–R3, top-left maze plot). The 6 maze plots show agent paths before (left, blue traces) and after (right, orange traces) the cooperative reward capture (see Section 2.4) indicated by the label to the left of the plots. Additional details are as described in the caption for Fig. 4.

We simulated the qEI-optimized NeuroSwarms model on both the Hairpin and Tunnel mazes (see Fig. 1B). Trajectory-trace plots for the Hairpin (Fig. 4, blue traces) depict the movement of each agent that contributed to reward capture throughout the simulation, up to the timestep at which cooperative capture of each reward goal was achieved. Likewise, trajectory traces in orange (Fig. 4) reflect the behavior of the reward-capturing agents after the reward had been captured. For example, the transition from swarming and goal-directed dynamics to post-capture exploration is depicted by the capture of Reward 3 (R3) in the third row of Fig. 4, in which a subset of agents converged on and captured R3 and immediately dispersed, thus permitting the search for and capture of subsequent reward goals. Agents recommenced exploration following reward-capture because NeuroSwarms relies on local, line-of-sight communication between agents, meaning that agent motion may not be influenced by nearby rewards if they are occluded by walls of the maze. The qEI-tuned swarms were able to quickly capture all five rewards on the Hairpin environment (*t* = 25.38 s), as shown in Fig. 4, whereas the original default parameters of NeuroSwarms—determined by hand-tuning as described in our previous work [34]—produced relatively slow reward capture (*t* = 41.02 s). Reward-capture speed using the default parameters was additionally exacerbated in the Tunnel maze (*t* = 175.42 s). In contrast, the qEI-tuned swarm captured all three rewards (Fig. 5) faster than the default swarm captured two rewards (*t* = 34.88 s). We attribute the worse performance of the hand-tuned default parameters to longer dynamical time-constants and thus slower behavioral responsivity. Thus, compared to manual parameter tuning for each maze environment, our Bayesian batch-optimization process (Section 2.3; Fig. 1A) with joint objective sampling (Section 2.4; Fig. 1B) was able to simultaneously, jointly, and efficiently discover distinct high-performing dynamical parameters for multiple mazes.

A key feature of our Bayesian optimizer is that the objective *indirectly* quantifies (i.e., as a ‘black box’ model) cooperative foraging without *directly* modifying NeuroSwarms’ underlying mechanisms. In general, this feature allows a task-dependent objective to evaluate multi-agent performance in collective tasks involving, e.g., social coordination or distributed consensus. In contrast to the regular but fragmented geometry of the Hairpin maze (Fig. 4), the Tunnel maze required the swarm to distribute through an irregular geometry to complete the foraging task (Fig. 5). Additionally, whereas agents were initialized at uniform random locations in the Hairpin maze, all agents in the Tunnel maze were initialized to points inside a small disc circumscribed within its Southwest quadrant. As a result, the agents rapidly capture R2 (Fig. 5, top row) and then split into subgroups to capture the remaining two rewards (Fig. 5, lower two rows). An additional challenge of the Tunnel maze is that R3 is initially visible to all agents and closer than R1, yet the tunnel constricts access to it. Conversely, R1 is initially visible and accessible, yet further away and partially occluded once agents have converged onto R2’s location. The fast capture of R1 (*t* = 5.46 s) vs. R3 (*t* = 31.78 s) reflects the characteristic time-scale differences between coordinated reward-approach trajectories and exploratory swarming trajectories, respectively. Comparing the pre-capture (blue, left) and post-capture (orange, right) trajectories for each reward (Fig. 5), the agents began using the large opening in the center of the map only once R2 and R1 were both captured. This behavioral transition suggests that exploration traded off with goal-directed exploitation by adaptively forming and regrouping subgroups of agents. Thus, distinct challenges presented by the Tunnel maze, in concert with our optimizer’s objective function definition (Section 2.4), may have induced collective behaviors that can flexibly adapt to diverse foraging problems.

### 3.4. Exploring the future parameter space

**Fig. 6.**
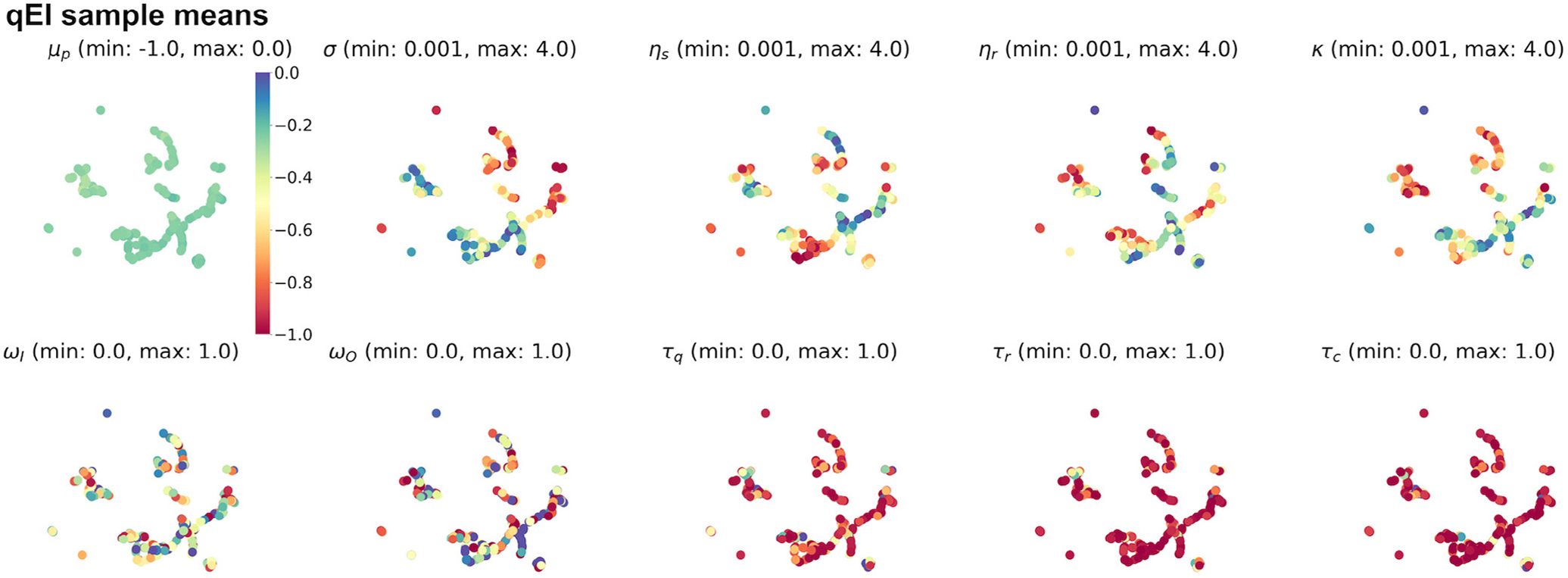
Anticipated future qEI-sampled parameter points. As in Fig. 3, a UMAP projection is shown across a series of plots: the top-left scatter plot assigns colors to each 2D UMAP point based on the colorbar to the right of the plot as indexed by the surrogate model’s expected posterior mean for each associated parameter point; the remaining *p* = 9 plots depict the same UMAP transformation except that the color of each point is mapped to the specified range (i.e., [min, max]) of the given NeuroSwarms parameter (cf. Table 1). A large batch of 500 qEI-based parameter samples is shown to facilitate visual inspection of the local structure of the trained surrogate model. For instance, these plots show that posterior sample means (top, left) have converged to similar high-performing values, and that most of the discovered system behaviors rely on short time-constants in the neural controller’s dynamics (viz., the prevalence of red data points in the three τ_*_ plots).

**Fig. 7.**
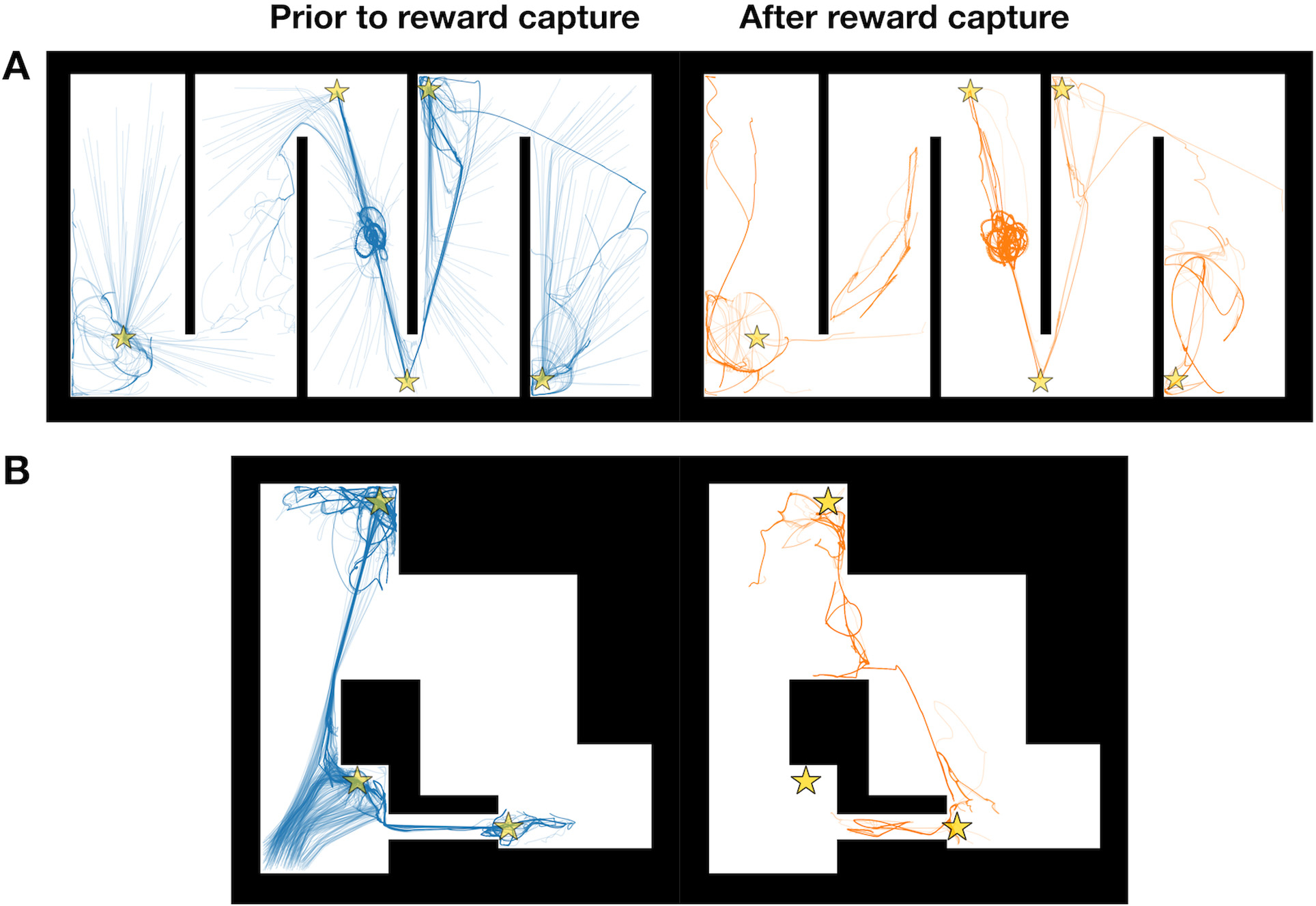
Example reward-capture trajectories from selected future qEI-sampled NeuroSwarms parameters. Pre-capture (left, blue traces) and post-capture (right, orange traces) pairs of trajectory-trace plots are shown relative to example reward-capture events from qEI-selected simulations in the Hairpin (*A*; cf. Fig. 4) and Tunnel (*B*; cf. Fig. 5) mazes. Parameters were selected for mid-range values (i.e., away from parameter range limits) from predictive (anticipated future) samples generated by the trained qEI-based surrogate model. Our Bayesian batch-optimizer naturally produces diverse output parameters that allow for the selection of distinct high-performing solutions and system behaviors, all of which have been equivalently constrained and guided by the high-dimensional shape of its task-dependent objective function.

Trained acquisition functions can be used to predict the performance of unobserved regions of the parameter space. To test predictive selection, we generated 500 samples from the qEI acquisition function and the posterior distribution of its trained Gaussian process surrogate model. The qEI sample means from the posterior (Fig. 6, top-left plot) were similar across most data points because qEI had adapted to parameter regions with the highest likelihood of utility improvement. As in the previous Section 3.3, we selected candidate points from these anticipated future qEI parameters to simulate in the Hairpin and Tunnel mazes, but we chose points that featured mid-range parameter values, i.e., whose vector elements were not at or near the range limits of the respective parameter (Table 1). In particular, we selected parameters where the time-constants were greater than the minimum of their ranges (1 ms), constituting a parameter regime that was distinct from clusters of qEI samples which minimized their respective time-constants in response to the time-pressure imposed by our objective function (Eq. (2)). We chose these points, with corresponding simulations shown in Fig. 7, to demonstrate the distinct behavioral solutions to the foraging task that can be discovered by the same acquisition function and associated surrogate model. Trajectory-trace plots of reward-capturing agents before and after rewards were cooperatively captured on the Hairpin and Tunnel mazes show that the selected parameters resulted in slower reward capture for the Hairpin (*t* = 47.44 s; Fig. 7A) and Tunnel (*t* = 66.96 s; Fig. 7B) mazes compared with the optimized parameters in Fig. 4 (Hairpin, *t* = 25.38 s) and Fig. 5 (Tunnel, *t* = 31.78 s). Additionally, the default parameters from Monaco et al. (2020) [34] entailed strong reward-approach exploitation (e.g., *κ* = 6.6), but weak swarming-based exploration (e.g., *σ* = 2.0). This combination of behavioral forces increased the time-to-capture for all five rewards. Thus, we attribute slow reward-capture to a combination of longer dynamical time-constant parameters and exploration–exploitation mismatches. Moreover, if the energy budget of agent locomotion (e.g., speed, turning, etc.) were to be taken into account by the objective function, a slower behavioral repertoire enabled by these parameter regimes could help to minimize energetic or inefficient navigational patterns.

## 4. Concluding remarks

Neuroscience-inspired learning and control methods have seen increased interest from robotics, artificial intelligence, and multi-agent control. Here, we presented a demonstration of exploring and visualizing the parameter space of a multi-agent model with complex dynamical behaviors using sample-efficient Bayesian optimization with Gaussian process surrogate models. We introduced an objective function for a spatial cooperative foraging task in NeuroSwarms simulations [34] to predict reward-capture performance across two distinct maze environments. Training the surrogate model was facilitated by the qEI and qNoisyEI acquisition functions. In particular, qEI was shown to guide optimizer trajectories towards parameter regions with high utility improvement, outperforming random sampling and manual tuning

By learning UMAP embeddings [57], we demonstrated visualization of 9-dimensional parameter points to identify and select high performing clusters of parameters. We illustrated the identification of parameters that generalized across environments by jointly evaluating the NeuroSwarms metacontroller in two distinct maze environments. Overall, our study serves as an example application of Bayesian optimization of complex multi-agent models to explore and select for complex behaviors like goal-directed spatial navigation in a system with distributed neural control.

As parameter size grows, the computational cost of the matrix inversions required to calculate updated Gaussian process parameters increases exponentially and eventually outweighs the gains in adaptive search efficiency provided by computing the acquisition function over the surrogate model to advance the sample trajectory [20]. This limitation on model dimensionality does not, in general, prohibit analysis of complex dynamics, particularly in systems of homogeneous particles, but it would reasonably detract the feasibility of Bayesian optimization for modeling systems with nontrivial heterogeneity in agent/particle behaviors. Within that moderate limit on model complexity—e.g., for *p* up to ~20—Bayesian optimization may facilitate adaptive and efficient computational exploration of dynamical parameter spaces, resulting in the identification of distinct and complex system behaviors.

Future work is needed to develop new controller models and critical spatial tasks to explore the capabilities of multi-agent objective functions that adapt efficiently to the characteristics of diverse environments (e.g., occlusive geometry, dynamic change, reward distribution, cue richness, etc.). We theorize that heterogeneous variation of swarm spatial structure and intertemporal coordination dynamics will be able to support a form of swarm metacognition that allows adjustment to the available goals in an environment, without initial knowledge of the goals or their locations. This approach could extend the flexibility of Bayesian optimization to operate in diverse environments and adapt efficiently to tasks with difficult or uncertain goals.

## Figures and Tables

**Table 1:** Tunable parameters that governed the spatiotemporal dynamics of the example NeuroSwarms model implementation [34]. ‘Range’ indicates the limits of the parameter subspace made available for Bayesian optimization. All other NeuroSwarms parameter values and constants were fixed at the defaults in Table 1 of Monaco et al. (2020) [34].

Name	Range	Description

*σ*	[10^−3^, 4]	Normalized interagent spatial scale
*κ*	[10^−3^, 4]	Normalized reward-approach spatial scale
*η_s_*	[10^−3^, 4]	Recurrent interagent learning rate
*η_r_*	[10^−3^, 4]	Feedforward reward-approach learning rate
*ω*_0_	[0, 1]	Baseline agent oscillation frequency
*ω_I_*	[0, 1]	Max. activation-based frequency increase
*τ_q_*	[0, 1]	Recurrent interagent time-constant
*τ_r_*	[0, 1]	Feedforward reward time-constant
*τ_c_*	[0, 1]	Sensory input time-constant
